# Techno-Economic Assessment of Whey Protein-Based Plastic Production from a Co-Polymerization Process

**DOI:** 10.3390/polym12040847

**Published:** 2020-04-07

**Authors:** Bushra Chalermthai, Muhammad Tahir Ashraf, Juan-Rodrigo Bastidas-Oyanedel, Bradley D. Olsen, Jens Ejbye Schmidt, Hanifa Taher

**Affiliations:** 1 Department of Chemical Engineering, Khalifa University, 127788 Abu Dhabi, UAE; bushra.chalermthai@ku.ac.ae; 2 Department of Chemical Engineering, Biotechnology and Environmental Technology, University of Southern Denmark, 5230 Odense M, Denmark; muta@kbm.sdu.dk (M.T.A.); jrbo@kbm.sdu.dk (J.-R.B.-O.); 3 Department of Chemical Engineering, Massachusetts Institute of Technology, Cambridge, MA 02139, USA

**Keywords:** whey protein, bio-based plastics, technical assessment, economic assessment, profitability, feasibility

## Abstract

Bio-based plastics, produced from natural and renewable sources, have been found to be good replacers to petroleum-based plastics. However, economic analyses have not been carried out for most of them, specifically those from whey. In this study, a techno-economic assessment of the industrial-scale production of plastics from whey protein is carried out considering two different scenarios: (1) low-cost dairy waste whey (DWP) and (2) purchased whey protein concentrate (WPC), as feedstocks, using SuperPro Designer software. Key economic indicators such as operating cost, capital investment, annual revenue, payback time, and return-on-investment (ROI), were analyzed. Sensitivity analyses of different parameters were performed to account for market fluctuations and other uncertainties, using Scenario 2 as the base case. Results showed that both scenarios have the capacity of producing over 3200 metric tons/year (t/y) (or 5.5 t/batch) of plastic. With the unit selling price of plastic set at $7,000/t, both the scenarios showed profitable outcomes with the plant’s payback time of 3.7 and 2.4 years, and ROI of 27.1% and 42.2%, for Scenario 1 and Scenario 2, respectively. Sensitivity analyses showed that the unit plastic selling price was the most sensitive parameter, followed by the amount of feedstock WPC, and the number of batches.

## 1. Introduction

Of all concerns related to waste management and its associated environmental impact issues, single-use plastics are now at the top of many agendas [1]. The increased awareness in the past decade has encouraged people towards using alternatives such as cloth bags for shopping and reusable bottles for drinking water. However, the effort is still lacking in many countries around the world, either because of limitation of alternatives, or of the individual’s own lifestyle and unawareness towards the problem. Of all the plastics produced, 60% of the wastes end up in landfills and the natural environment, including oceans [2,3,4,5]. Alternatives to the synthetic, non-biodegradable, and fossil-derived plastics have been studied and commercialized to minimize their environmental impact. Although plant-based plastics are biodegradable, their use competes with the food industry and hence interferes with food security. Therefore, second-generation feedstocks, i.e., biomaterials which are by-products or wastes such as corn stover, wheat straw, miscanthus, hardwood, and short cotton fibers were therefore investigated [6,7]. Currently, the global production capacity of bioplastics is about 2.1 million metric tons (t) [8], which are used for different applications such as packaging, consumer electronics, agriculture, toys, and textiles. Packaging contributes to the majority of the bioplastics produced, at 53%, or 1.14 t in the bioplastic market in 2019 [8].

Second generation feedstocks may also be obtained from dairy industry. Whey liquid is usually obtained as a by-product in the cheese-making and yoghurt industry. Prior to the 1920s, whey obtained from cheese production were conventionally discarded in waste streams, thereby harming the aqueous environment due to their high biochemical oxygen demand (BOD) and chemical oxygen demand (COD) levels [9,10]. The utilization rate of whey was reported as 75% in Europe, and less than 50% in the rest of the world [11]. In 2015, the European Dairy Association (EDA) also estimated that 6 million out of 9 million t (which accounts for 67%) of whey is processed from cheese and caseinates in the EU (European Union) [12]. As awareness in the last century increased, whey-rich streams were treated (pasteurized, centrifuged, and concentrated) and used to produce functional food products such as whey protein concentrate (WPC), with protein content ranging from 30%–85% [13,14,15]. Due to their nutritional values, WPCs are widely available in the market, especially as body-building food supplements. The monthly production of WPC in the U.S. was found to reach 18,600 t in 2018 [16]. The sales price for WPC lies in the range of $25/kg to $40/kg [17]. It is worth mentioning that large cheese making industries have processing units within their facilities that can convert whey into value-added ingredients, such as whey protein powder. However, this is not possible in small industries. For example, daily production of 3,785 L whey is not worth picking up for an industrial processor, as they need closer to 30,000 gallons (113,562 L) per pick up [18], resulting in huge amounts of whey wastes. In fact, some yoghurt producers have to pay farmers to pick up their waste whey liquid at $300 per 6000 gallons (22,713 L) truckload to use them as feed and fertilizers in their farms [19]. However, some farms in Italy store liquid whey and sell them at a rate of €18.88 ($21.21)/t [20]. This waste whey have thus been valorized to make high-value products, such as plastics [21,22,23], replacing the conventional, environmentally-harmful petroleum-based plastics. 

Although several studies have been carried out to produce bioplastics from whey proteins, to the best of our knowledge, no previous works on assessing the economics of the production of whey protein-based plastics were completed. The techno-economic assessment is used in several research projects to analyze the technical and economic aspects of a process envisioning the bulk processing of raw materials for the bulk production of marketable products. In recent years, the techno-economic assessment was performed for several waste-to-energy and waste-to-high-value product plants. These assessments were performed for plants that utilized municipal solid wastes [24,25], food-wastes [26,27], and lignocellulosic biomass [28,29,30]. In these studies, the biorefinery concept was applied, where high-value products such as biochemicals and biofuels were produced from renewable materials and not from fossil fuel resources [31]. More specifically, waste from the dairy industry, such as whey wastes, were also analyzed based on their techno-economic aspects, to produce useful whey protein and lactose products [15,32]. A study carried out by Bochenski et al. [33] analyzed the techno-economic aspects of plastic-production plants from modified algae proteins using copolymerization process with copolymer. 

This study analyzes the technical and economic aspect of upscaling the plastic production process from whey proteins. The copolymerization of modified whey proteins was carried out with synthetic copolymer poly(ethylene glycol) methyl ether methacrylate (PEGMA) and some approximations derived from literature. Two main scenarios using different sources of feedstocks are considered in this work; namely using (1) waste liquid whey obtained directly from the dairy waste stream (DWP), and (2) whey protein concentrate (WPC, powder in nature) purchased from market. The second scenario is considered as the base case scenario for sensitivity analyses. The profitability of the plant is discussed from the results of the plants’ costs, payback time, and return-on-investment (ROI). 

## 2. Materials and Methods 

### 2.1. Software

SuperPro Designer v.10 ^®^ (Academic Ed.,Scotch Plains, NJ, USA) was chosen to model the plastic-production plant. The software consists of several unit procedure models that can be used to design the upscale version of the experiments conducted at laboratory scale. Its main features, including extensive chemical component and mixture database, extensive equipment and resource databases, equipment sizing and costing, and thorough process economics, make it advantageous to be used for techno-economic analysis. The unit operations relevant to this study are described in Section 2.2 and Table 1. 

### 2.2. Process Description

**Figure 1:**
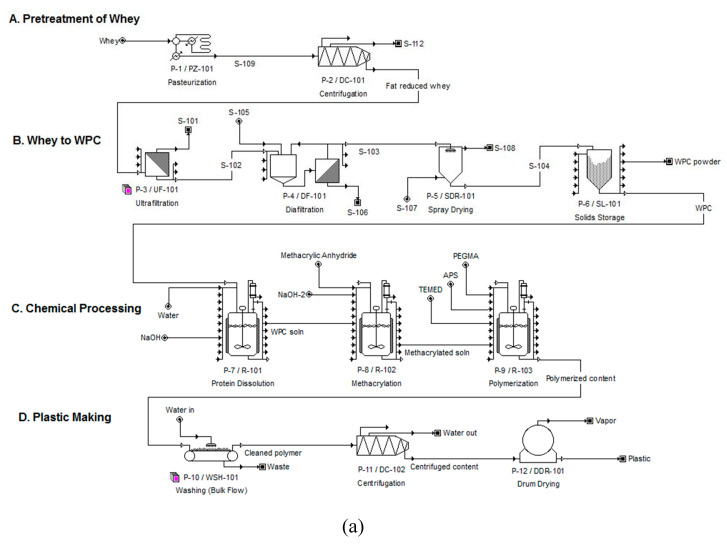

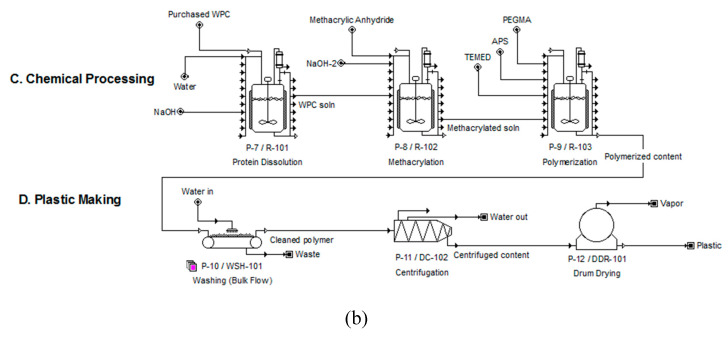
Process flow diagrams of bioplastics production using SuperPro Designer ^®^ for (**a**) Scenario 1, and (**b**) Scenario 2.

For the assessment of an alternative process to produce bioplastics, two scenarios were considered: Scenario 1; the feedstock of the plant is DWP, and Scenario 2; the feedstock is purchased WPC. The simulations were considered for batch processes, based on the experimental procedures published earlier [23] which were done in batches, with maximum annual operating time of 7920 h (330 days). The processing of Scenario 1 was divided into four major steps: A) pretreatment of whey (P-1 to P-2), B) production of WPC from whey (P-3 to P-6), C) chemical processing (P-7 to P-9), and D) plastic making (P-10 to P-12), as indicated in Figure 1a. As the feedstock for Scenario 2 was WPC, it consisted only of the last two steps C and D (Figure 1b). The operations carried out in each of the unit procedures from P-1 to P-12 are described as follows, with equipment and unit operation details described in Table 1.

#### 2.2.1. Step A: Pretreatment of Whey

##### P-1/PZ-101 Pasteurization

Pasteurization, a heat treatment process, was considered for the removal of bacterial impurities from the waste whey. Waste whey could have also been obtained from milk that was already pasteurized, but it was considered here to help in reducing early fouling of the waste whey during the processing. Since different whey protein denatures at different temperature (ex: β-lactoglobulin at 78 °C, α-lactalbumin at 62 °C, and bovine serum albumin at 64 °C), the pasteurization temperature of 61 °C was considered in the simulation, so that no denaturation of protein would take place. Pasteurization for 15 s was considered, which is the usual time for pasteurization of milk [34]. The whey is then cooled down to 30 °C and transferred to the next step for centrifugation. 

##### P-2/DC-101 Centrifugation

In this procedure, large molecules such as fats from the waste whey are separated from the liquid sample. Centrifugation is a common separation operation in the chemical, biochemical, food, and environmental industries [35,36]. The solids components removal is targeted at 60% and that the remaining amounts are filtered out in the next step of separation.

#### 2.2.2. Step B: Production of WPC from Whey

##### P-3/UF-101 Ultrafiltration

In ultrafiltration (UF), the filtration of solutions or suspensions take place under pressure through a semipermeable membrane, which allows only the solvent and small molecules to pass through while the larger molecules are retained [37]. It is worth mentioning that UF is a crucial step in the production of WPC as it is used to remove large molecular weight solutes and particulate components, thereby concentrating protein solutions and separating proteins from the low molecular weight solutes [13,38,39]. The rejection coefficient (RC) was set at 0.95 for proteins, the concentration factor (feed to retentate mass ratio) at 20:1 (it can range from one to five folds [37]), and filtrate flux left at SuperPro Designer’s default value of 20 L/m^2^/h (which is in the typical range of 20–50 L/m^2^/h [37,40].

##### P-4/DF-101 Diafiltration

Ultrafiltration is usually accompanied by diafiltration, hence called UF/DF, in downstream processing for product concentration. Diafiltration is a dilution process that helps to remove more permeating components or fats, carbohydrates and minerals from proteins, thereby enhancing the yield and purity of the protein separated [37]. The rejection coefficient for proteins was set at 0.95. The filtration time was left as default at 4 h, with filtrate flux also as default, 20 L/m^2^/h.

##### P-5/SDR-101 Spray Drying

After macromolecule separation, the whey protein solution is then spray dried in order to remove the water content and powder whey protein concentrates are obtained. The amount of water removed, through evaporation, was set at 93%, so that the final powder product would retain moisture of about 10%, which is higher than the average moisture content of protein powder of 6% [41], to account for lower efficiency of the product. Such water content of the sample is still acceptable since the WPC here is targeted for plastic making, not for food applications.

##### P-6/SL-101 Solids Storage

The WPC obtained from spray drying was then stored in a silo for bulk storage for 1 h. The amount of WPC obtained from 100 t of treated whey was found to be 1.04 t (P-1 through P-6). Therefore, 1 t of the WPC was transferred from the storage tank for the plastic manufacturing, whereas the remaining amount of 0.4 t was assumed to be sold cheaply at $0.001/t as WPC powder itself. The reasons for dividing this procedure into two output streams are (1) to account for errors that can occur in each batch in case that the full efficiency of the plant is not realized and that less than 1.04 t of WPC may be produced in some batches, and (2) only 1 t is needed to run Scenario 2 (P-7 onwards), for simplicity of calculations and analysis of data. 

#### 2.2.3. Step C: Chemical Processing

##### P-7/R-101 Protein Dissolution

In this procedure, 1 t of WPC powder was dissolved in 10,000 L of water, making 10 wt % protein solution, as per the experimental design described in Chalermthai et al. [23]. The pH of the solution was also adjusted by adding 100 L of 10 M NaOH solution (i.e., 1:100 ratio of NaOH:protein solution) to achieve a pH of about 8 to 9. This helped ensure the protein precipitation not to occur when the pH reduces to near its isoelectric point in the range of 4 to 5. 

##### P-8/R-102 Methacrylation

The dissolved and pH-adjusted protein solution is then transferred to the methacrylation reactor (P-8) where 50 L of methacrylic anhydride was added (as per 50 µL/g of protein concentration condition). To ensure the maximum extent of the reaction to take place, the solution was left to agitate for 7 h. Methacrylic acid, a by-product of such reactions, as described in Chalermthai et al. [23] would lower the pH of the solution, therefore, 125 L of 10 M NaOH solution was added to maintain the pH in range of 9 to10, thereby avoiding protein precipitation. 

##### P-9/R-103 Polymerization

The methacrylated protein solution was then transferred to the polymerization reactor (P-9). To satisfy the protein:copolymer ratio of 30:70 (since this is the ideal concentration found earlier [23]), PEGMA (copolymer), ammonium persulfate (initiator) and TEMED (catalyst) were added at 2300 L, 92 L and 4.6 L, respectively. The polymerization reaction (described in [23,42]) lasted for 1 h and was then left at ambient condition for 2 h. 

#### 2.2.4. Step D: Plastic Making

##### P-10/WSH-101 Washing

The purpose of the washing procedure is to remove the unreacted monomer and other chemicals and to condition the plastic produced [43] since these impurities could lower the efficiency of the centrifugation and drying steps, thereby affecting the purity of the final product as well. The impurities were washed off as aqueous wastes, which were assumed to be charged by other waste treatment utility at $50/t (assumed to be about the same price as solid hazardous waste treatment cost in Abu Dhabi, UAE) [44].

##### P-11/DC-102 Centrifugation

Water from the polymerized content was then removed in two stages in P-11 and P-12. In P-11, water content in the solution was reduced so that the final product contained 40% water. 

##### P-12/DDR-101 Drum Drying

Further amounts of water were removed through drum drying where 85% of the water evaporation took place. This resulted in a dried polymer with 90% polymerized content and 10% moisture. Drum drying was selected as it is the most universal unit procedure for this step of operation. Dried polymer solid was scraped off the rolls as they slowly revolved and cut off by knife from internal surface. Depending on the final application of the plastics produced, different types of shapes may be formed. Hence, this procedure was selected here for general application assumed in this model.

### 2.3. Feedstocks 

Two main feedstocks were considered in the simulations for plastic production, based on the two different scenarios, namely Scenario 1 where the feedstock is waste whey from dairy or cheese-making industry (DWP) and Scenario 2, where the feedstock is whey protein concentrate (WPC) purchased from market. The reason for choosing these two scenarios in this study is because the results obtained from the model plastic-production plant can be implemented in any country around the world. While the majorly cheese-producing countries may have a lot of dairy farms, a lot of waste whey may be collected more easily for further processing into high-value products. However, other places may make use of commercially-available WPC that are expired and unfit for human consumption so that these WPC do not go to waste and be used for plastic production. The DWP was considered to be obtained for free from the dairy waste stream, assumed to be closed to the plant’s location (hence no additional transport cost), where its amount was set at 100 t, so that it is efficient to utilize this waste amount [18]. For the WPC, on the other hand, the bulk price was used, which is $6000/t [16]. The composition of whey waste from dairy industry is taken as the averages of values found in literature [10,45,46], i.e., 4% carbohydrates, 4.5% fats, 1% minerals, 1% proteins and 89.5% water. The composition of the WPC was taken as the result calculated from the processing of the whey to WPC in the simulation and was the closest to the values in literature [13,47], namely 5.1% carbohydrates, 4.9% fats, 1.3% minerals, 78.7% proteins, and 10% water.

### 2.4. Other chemicals

Bulk prices of the chemicals used were estimated as average values from different suppliers on www.alibaba.com, as well as values found in literature [33]. These are compared to the Sigma Aldrich prices to check for consistencies. The costs of the materials and chemicals used in the SuperPro Designer simulation per metric ton are: Ammonium persulfate $600, methacrylic anhydride $8000, copolymer PEGMA $3000, sodium hydroxide $300, and catalyst TEMED $4200.

### 2.5. Economic Evaluation

The utility prices were taken from literature and/or from SuperPro Designer’s default values. The costs are: electricity $0.1/kWh, steam $12/t, and water $2.14/t (software default values, close to Abu Dhabi’s water and electricity costs [48]). 

Equipment sizes and prices were determined using built-in models in SuperPro Designer. The design of the plant is based on literature for typical whey protein and plastic-production plants [15,38,43]. The capacity of the plant is based on the amount of the materials that are processed in the plant. Other components of the investment cost related to the plant were estimated based on the total equipment purchase cost in SuperPro Designer or from well-established values found in literature, which are: piping (31%), instrumentation (28%), electrical facilities (10%), buildings (22%), and construction (34%) (all from SuperPro Designer default values); and insulation (3%), yard improvement (10%), engineering/supervision (25%), and auxiliary facilities (40%) (all from [49]). Other costs such as the income taxes (40%), interest rate (7%), inflation rate (4%) and other miscellaneous costs are included in the calculation using the default SuperPro Designer values. The labor wage is calculated to be $46/h, based on the default basic rate of $20/h. 

For the analysis of the results, the economic parameters considered include the total capital investment of the plant and the annual operating costs. The total capital investment refers to the fixed costs that are associated with a process. Detail calculation of the costs are given in the Supplementary Material. 

Besides the costs, the revenues generated from the process will result in profits (or loss) of the plant. Intuitively, to be able to gain profits, the unit price of the plastic sold must be greater than the unit price of the production. Hence, depending on the amount of the plastics (production volume) that can be sold in a year, and the total capital investment (total fixed costs), the minimum selling price (MSP) can be calculated and used as the selling price of the product. The revenues generated from the plant are also considered and these are calculated directly from the unit production revenue of the plastic defined by the user and the amount of the plastic produced annually. The measure of profitability is reported as payback time and return-on-investment (ROI). The equations associated with these parameters are provided in the Supplementary Material.

## 3. Results and Discussion

The designed chemical process, shown in Figure 1, was developed to analyze the techno-economic aspects of upscaling the lab-based plastic production process to industrial scale in terms of different profitability measures mentioned earlier. Full economic details are provided in the Supplementary Material.

### 3.1. Economic Results

By starting with 100 t of waste whey and 1 t of WPC in Scenarios 1 and 2, respectively, process simulations showed that both plants have the capacity to produce over 3200 t/year of plastics. The total capital investment for Scenario 1 was found to be higher and reached $33.56 million, compared to $19.13 million for Scenario 2. The unit production costs were $3850/t and $3680/t for Scenarios 1 and 2, respectively. The economic analysis results are shown in Table 2 (more details on the annual operation costs and profitability analysis are provided in the Supplementary Material). In each batch, about 5.5 t of plastics are produced and sold at $7000/t, calculated from the MSP (Supplementary Material Table S1). The unit selling price of plastic was set at $7000/t which is lower than the MSP for both the scenarios, to account for uncertainties of the production; like equipment and machines breakdown, lower annual hours of operation or fewer batches per year. In addition, this was the reported price set for algae-derived plastics using the same methods of methacrylation and polymerization [33]. The price for the whey protein-based plastic in this study should be greater, since it is postulated to be of better quality than the algae-based plastics, and hence, sensitivity analysis is done on a range of prices that the plastic obtained can be sold at too.

As illustrated in Table 2, the total capital investment was found to be $33.56 million for Scenario 1 and $19.13 million for Scenario 2. As expected, the capital investment for Scenario 2 was lower than the one in Scenario 1 since only the second half of the plant of Scenario 1 is reflected. This is considered to be normal for a plant of such capacity, as other plants that are modeled by Silva et al. [15] to produce WPC (our mid-product) from whey are in the range of $7 to $26 million. However, since our plant’s final product is plastic of over 5 t, the capital investment is higher in Scenario 1 with more unit procedures in the processing of the plastic. The revenues generated from the plastic stream in Scenario 2 was found to be $22.59 million/y. This is $53,000 greater than the revenues generated in the Scenario 1 ($22.54 million/y), which is attributed to the fact that Scenario 1 has more equipment and processing steps and hence the unit production cost was higher. The net profit in Scenario 1 was $9.09 million/y, whereas it was found to be $8.08 million/y in Scenario 2. This amount of profit for both the scenarios makes the plant very viable to be operated. In Scenario 2, the unit production cost was lower, at $3680/t MP, compared to that of $3850/t MP in Scenario 1. Compared to literature, this cost is considered to be feasible. For example, a study done by Mudliar et al. [50] showed that the unit production cost of biopolymers like polyhydroxybutyrate (PHB) from activated sludge was $5380/t - $11,800/t PHB. Another study showed a lower price, where the unit production cost was 1.4–1.95 euros/kg PHB (or $1560–2170/t PHB) [51]. Another study on the production of bioplastic Poly(3-hydroxybutyrate) (P(3HB)) from sugarcane bagasse showed that the unit production cost was $3440/t P(3HB) [52], which is close to the value obtained in this study.

In Scenario 2, the unit production revenue is $7000/t MP, which is close to that in Scenario 1 since the same amount and quality of the WPC is used, but no extra WPC is sold separately. Scenario 2 showed a very high ROI of 42.24% with a reduced payback time of only 2.37 years, compared to 3.69 years in Scenario 1. These values are much lower compared to the plastic produced from algae proteins using the same copolymerization process which has a payback time of 8.4 years [33]. These values show that the economic return is very positive and that the project is very profitable to be operated. 

**Figure 2:**
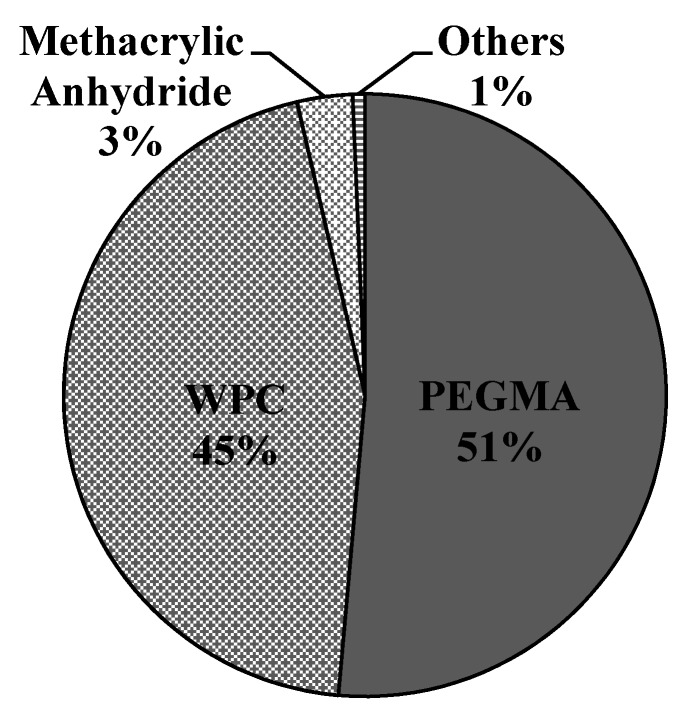
Annual materials costs contribution.

In Scenario 2, the total materials cost annually was found to be $7.88 million, which contributed to 66% of the annual operating cost (Supplementary Material: Table S2). The other costs such as facility-dependent, utilities and labor contribute to the annual operating cost on a lower extent. The materials that contribute to the major portion of the costs was the copolymer PEGMA (51%), followed by WPC (45%) and methacrylic anhydride (3%). This is similar to the results found in [33], where the price of PEGMA contributed the most, followed by methacrylic anhydride and proteins. Figure 2 illustrates the percentage contribution of each material in the annual cost. As can be seen, the amount of PEGMA contributes to the major portion of the plant because the proportion of protein:copolymer used was 30:70, i.e., the PEGMA is 2.33 times the amount of WPC used. Therefore, even if the price of PEGMA is reduced to half of that of the WPC, the amount is more than double, so the annual cost of PEGMA is higher. The price of WPC and methacrylic anhydride also contributed to the major portions of the materials consumed. The amount and prices of these three materials were therefore considered in the sensitivity analysis. 

It was found in our previous study that the polymers made from higher protein/PEGMA (or, WPC/PEGMA) ratio of 30:70 had significantly better tensile strength than the one at 20:80, by about 1–2 MPa [23], hence we chose to model with this proportion originally. It is worth observing the economic effects here though, in case the plastic is of lower quality. To consider the potential changes in the copolymerization effect of the produced plastic, a different proportion of protein/PEGMA of 20:80 condition was also tested. The comparisons of each scenario’s results on the payback time, ROI, total capital investment, annual operating cost, and annual revenues are as displayed in Table 3. With lower protein/PEGMA ratio (20:80, compared to the originally modeled 30:70), the payback time increased from 3.69 to 5.56 years for Scenario 1, and from 2.37 to 3.85 years for Scenario 2. The ROI also significantly reduced from 27.1% to 17.99% for Scenario 1, and from 42.24% to 25.95% for Scenario 2 when the protein/PEGMA ratio changed from 30:70 to 20:80, respectively. However, the total capital investment, operating cost, and the revenues, do not change significantly. Hence, from these results, it is proved that it is definitely more feasible to produce plastic at a 30:70 protein/PEGMA ratio. It is important to note that other proportions, such as a protein/PEGMA ratio of 40:60 and 50:50 were not considered because these are unrealistic, as polymers with good tensile strengths could not be produced at those concentrations. Hereafter, further sensitivity analysis is completed on other parameters using the 30:70 protein/PEGMA as our base case scenario.

For further sensitivity analysis, the baseline considered here is Scenario 2, where whey protein-based plastics are produced from purchased WPC powder. It was found that for the production of 3200 t/y of plastics, a total purchase prices of $3000/t of PEGMA and $6000/t of WPC would be required, which would result in 2.37 years to re-pay the amount invested. This would also require an annual operating cost of $11.9 million and capital cost of $19.1 million, resulting in a revenue of $22.6 million. The ROI in the base case was found to be 42.24%. In such case, total of 590 batches of production was considered per year. This is because the plant is assumed to be operational for a maximum of 7920 h (330 days per year). This leaves one month of maintenance, and other repairing works that may need to be completed. The sensitivity analysis was performed based on this baseline case by varying at least 25% decrease/increase in selected parameters.

### 3.2. Sensitivity and Parametric Analyses

The sensitivity analysis was performed to find the parameters that significantly affect the profitability of the plant, and to account for price fluctuations in the market due to technology development, availability of raw materials and other resources, and other aspects. Since some material costs were taken from the bulk price obtained from different sources while some others were assumed, the analysis was carried out to cover for any changes in the results. Moreover, some internal parameters, like processing techniques and equipment capacity, can all influence the outcome of the plant’s operating costs and revenues. The reference case of Scenario 2 is used for the analysis, since the feedstock WPC is more widely available around the world rather than liquid waste whey from dairy industry. In the analysis, all parameters were kept constant as defined by the base case scenario, and only the parameter under analysis was adjusted to reflect the changes in the payback time, operating costs, and revenues. 

**Figure 3:**
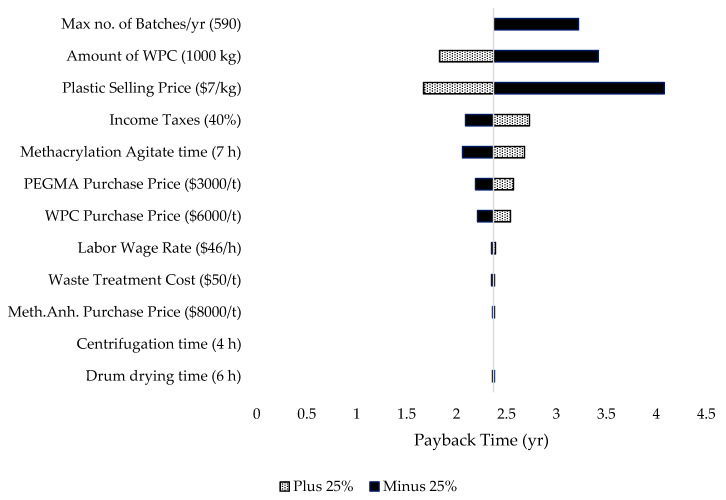
Tornado chart showing the sensitivity of payback time for different important parameters (the base case values for each parameter are displayed in brackets). Filled bars represent increase of 25% compared to their base case values whereas patterned bars represent the reduction of 25% compared to the base case values. The mid-line is the payback time for the base case value, i.e., 2.37 years.

The tornado chart (Figure 3) demonstrates the relative changes that may take place when input variables are changed in values. Data presented are for the parameters that are sensitive by comparing with other parameters when their values (prices, duration of equipment processes, etc.) are increased or decreased by 25%. The parameters that are the most sensitive are then picked for further analyses in order to explore more range of possible options to maximize the techno-economic efficiency. 

As can be observed in the tornado chart, the parameter that is the most sensitive is the plastic selling price. By reducing the selling price of the plastic by 25%, i.e., to $5250/t, the payback time increased to 4.08 years, compared to 2.37 years with the selling price of $7000/t in the base case. Moreover, the scale of the plant, determined by the number of batches the plant, runs per year and the amount of WPC feedstock used also affects the payback time of the project quite significantly. Meanwhile, other parameters like the labor wage rate, the waste treatment costs, and duration of some long processes like centrifugation and drum drying do not affect the economics (payback time) of the project. Hence, these parameters will be rejected for further economic analyses. Further analyses on sensitive technical and economic parameters are done on two main categories: internal parameters (related to the plant’s processes), and external parameters (such as prices of feedstock and chemicals).

#### 3.2.1. Internal Parameters

Internal parameters refer to those that are relevant to the plant’s process and design, i.e., production scale such as number of batches and amount of feedstock, selling price of product, and operation time of a process. 

##### Number of Batches

**Figure 4:**
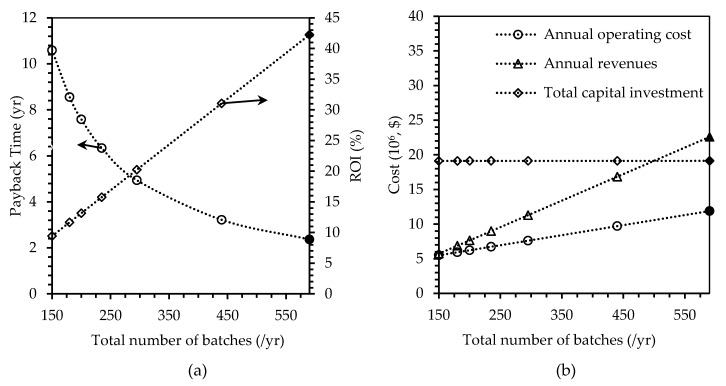
Effects of changing number of batches per year, on (**a**) payback time in years and return on investment (ROI) in percent, and (**b**) annual operating cost, annual revenues, and total capital investment in U.S. dollars. Filled markers represent results of the base case scenario.

A total of 590 batches of plastic production was considered in the baseline case. However, if the plant was operational for only 4 batches a week, and only 45 (out of 52) weeks a year, there would be only 180 batches per year, which is more than three times deduction in the production scale. In this case, the payback time of the plant would increase to 8.56 years, compared to 2.37 years in the base case. ROI was found to decrease by 72% to 11.68%, and the revenues by 69% to $6.89 million/y. The operational cost was reduced by half, from $11.89 million/y in the base case, to $5.94 million/y. The total capital investment was not affected greatly (only $5000 difference between the base case of 590 batches and the assumed case of 180 batches), but the operational cost was reduced by 50% from $11.89 to $5.94 million/y. To keep the payback time to below five years, the minimum number of batches should be at least 50% of 590 batches, i.e., 295 batches (payback time of 4.94 years). For a range of different payback times, ROI, total capital investment, operating costs, and revenues, the results are as shown in the graphs in Figure 4.

##### Amount of Feedstock

**Figure 5:**
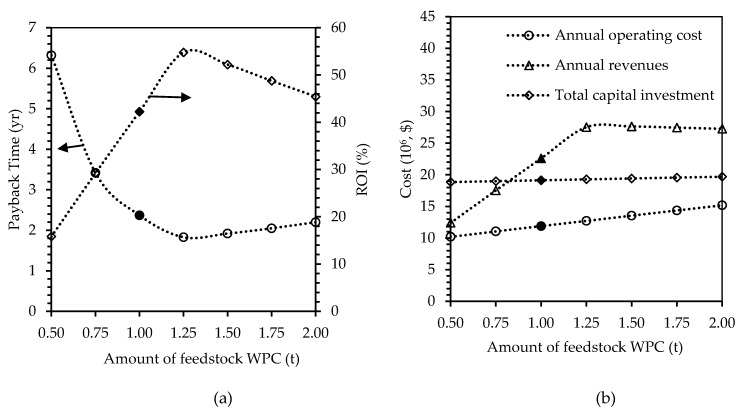
Effects of changing number of amounts of feedstock whey protein concentrate WPC, on (**a**) payback time in years and ROI in percent, and (**b**) annual operating cost, annual revenues, and total capital investment in U.S. dollars. Filled markers represent results of the base case scenario.

In the baseline case, the amount of feedstock WPC used is 1 t. Figure 5 shows the effect of increasing the amount of WPC by 25% (from 1 t to 1.25 t) on the five selected economic indicators. It was found that the payback time got reduced to 1.83 years, and ROI increased to 54.78%. The revenues generated by the increase of raw material amount also increased and reached $27.53 million/y, which is 21% higher than the baseline value. This shows that with this amount of feedstock, the plant would have the maximum attainable profit in a short period of time. Therefore, an amount of 1.25 t of WPC, which produced 6.72 t of plastics per batch, or 3965 t/y, would be optimal to be used in such plastic-production plants. On the other hand, if the amount of the WPC is reduced by half to 0.5 t, then the payback time increased to 6.32 yrs, which is over a three-fold increase. The ROI was also reduced three-fold to 15.82%. This shows that the downscaling of the plant by reducing the amount of feedstock has greater impacts on the profitability compared to when upscaling the plant size. The amount of WPC used is therefore a crucial factor as it impacts the economic outcomes of the plant in different ways depending on the increase or decrease in the amounts of raw materials used. Increasing the amount of feedstock would also require bigger equipment, which would affect the results differently.

##### Selling Price of Plastic Product

**Figure 6:**
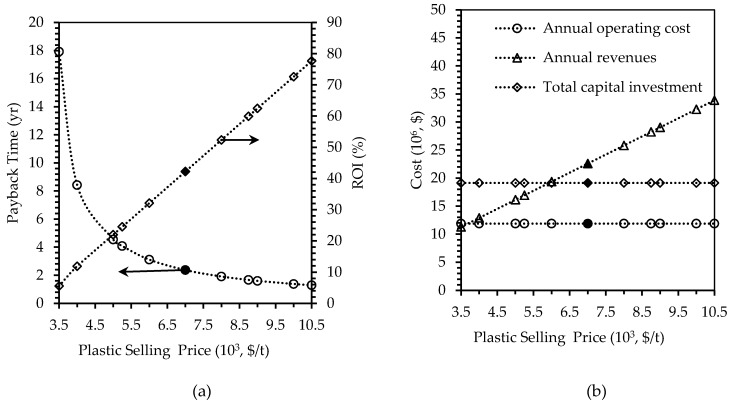
Effects of changing plastic selling price, on (**a**) payback time in years and ROI in percent, and (**b**) annual operating cost, annual revenues, and total capital investment in U.S. dollars. Filled markers represent results of the base case scenario.

The base case scenario assumed the produced plastic to be sold at $7000/t, which is the same as the price of the plastic obtained from algae biomass [33]. However, due to the better mechanical and thermal properties of whey-protein plastics, confirmed in our previous work [23], they can be sold at higher prices. Sensitivity analysis was carried on the prices ranging from -50% to +50% of the baseline price and the results are shown in Figure 6.

By halving the unit selling price of the plastic to $3,500/t, the payback time increased almost exponentially from 2.37 to 17.92 years (an increase of 656%). This would make the project very unfeasible as no profits can be obtained in a shorter period of time. The ROI was also very low and reduced from 42.24% to only 5.58% (reduced by 87%). The revenues decreased by half from $22.59 million/y to $11.29 million/y. This is huge loss since the total capital investment and the operating costs were maintained at the same amount of $19.13 million and $11.89 million/y, respectively. Hence, this confirms that the plastic selling price is a very sensitive parameter and that just a small change can affect the profitability of the plant significantly. Therefore, it can be concluded that the price of the plastic should not be sold for less than $5000/t in order to maintain the payback time of less than five years. On the other hand, increasing the selling price to $10,500/t instead of $7500/t would result in a payback time of 1.29 years, which is about one year less than the baseline case of 2.37 yrs. The ROI would also increase to 77.67%, which is profitable to the plant. The revenue would also increase from $22.59 million/y to $33.89 million/y. Given the mechanical and thermal properties of the plastics, it is very reasonable to charge them at about $10,500/t or even more, which could definitely provide great profits to the plant. 

##### Process Time

**Figure 7:**
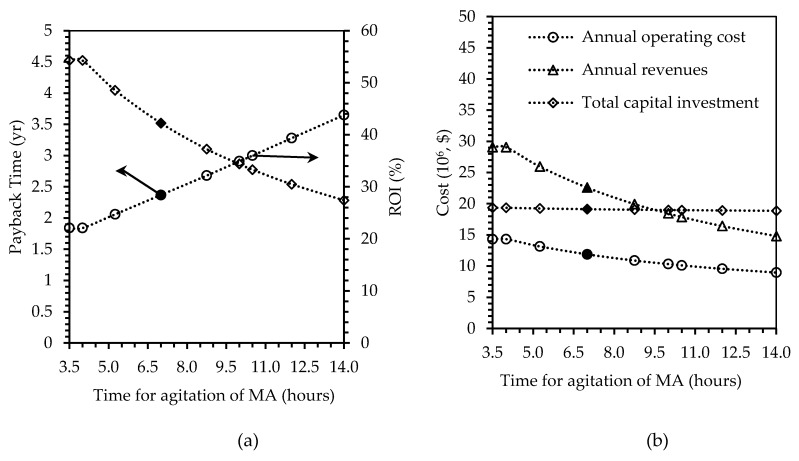
Effects of changing methacrylation agitation time, on (**a**) payback time in years and ROI in percent, and (**b**) annual operating cost, annual revenues, and total capital investment in U.S. dollars. Filled markers represent results of the base case scenario.

The methacrylation level of the proteins to be used for polymerization is very crucial as it could affect the properties of produced plastics [42]. Therefore, changes in the contact time between the protein solution and the methacrylic anhydride (MA) were considered in the sensitivity analysis. The degree of methacrylation is therefore dependent on that mixture time which can range from 2 to 24 h. In order to observe the economic variations, the methacrylation time was changed in a range of −50% to +100% of the base case scenario of 7 h. Figure 7 shows the effect of changing the time on selected indicators. It was found that doubling the contact time to 14 h resulted in increased payback time from 2.37 to 3.65 years and decreased ROI from 42.24% to 27.41%, which is about a 35% deduction. Similar rate of deduction was also found in the revenues, which decreased from $22.59 million/y to $14.82 million/y. Deduction in capital investment and operating cost were found to be insignificant, compared to other indicators. Hence, it is important to note that this process of methacrylation may not only affect the final properties of the materials, but also the economics of the plant itself.

##### Selling Price of Plastic and Amount of WPC

**Figure 8:**
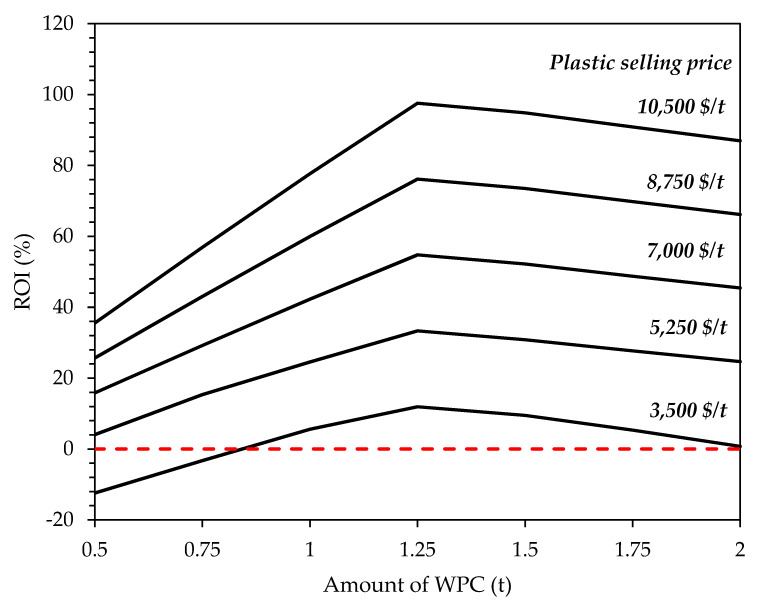
Effects of changing amount of WPC at different plastic selling prices on the ROI (%). Red dashed line represents ROI at 0%.

As discussed earlier and shown in Figure 3, the amount of WPC and the selling price of plastics were the two most significant parameters. This was based on the single factor effect on the parametric sensitivity analyses. Although this highlights the parameters that may significantly affect the profitability of the process, it does not account for any interaction between process parameters. Full scale multivariate sensitivity analysis is out of the scope of this study as the process simulator SuperPro Designer does not have this functionality. To account for parameters interaction, a two-dimensional sensitivity analysis of the two most sensitive parameters was conducted to analyze the combined effect of the amount of WPC and selling price of plastics on the ROI. The amount of WPC was varied from 0.5 to 2 t with a step size of 0.25 t and plastic selling price from 3,500 to 10,500 $/t with a step size of 1750 $/t, giving totally 35 distinct combinations of these two parametric effects. Simulation was run at the 35 distinct input values and ROI was calculated. The results are shown in Figure 8. As can be seen, the ROI was found to be less than zero only when the selling price of 3500 $/t and amount of WPC of less than 1 t were considered. The results also indicated that the maximum ROI can be reached at plastic selling price of 10,500$/t, considering 1.25 t of WPC. This shows that under the current parameter settings and economic assumption, the amount of WPC at 1.25 t is the optimum size for the plant.

#### 3.2.2. External Parameters

External parameters refer to those that are external to the plant’s process and design, such as prices of the chemicals and feedstocks used.

##### Purchase Price of PEGMA

**Figure 9:**
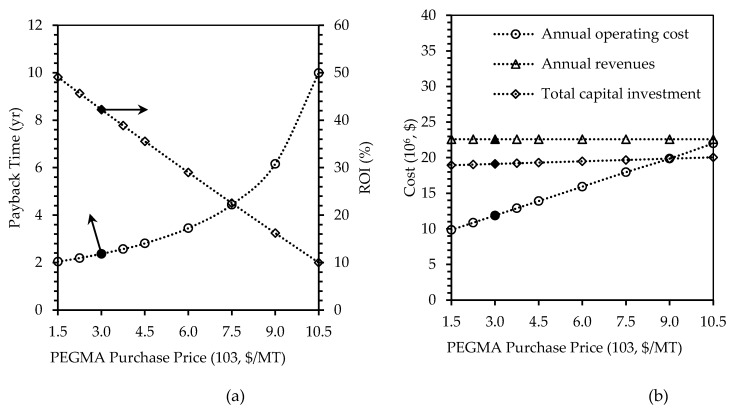
Effects of changing PEGMA purchase price, on (**a**) payback time in years and ROI in percent, and (**b**) annual operating cost, annual revenues, and total capital investment in U.S. dollars. Filled markers represent results of the base case scenario.

The tornado chart (Figure 3), showed that the PEGMA purchase price was sensitive to the overall profitability of the project. This is because 51% of all the material costs is contributed by PEGMA indicated in Figure 2. Figure 9 shows the effects of the variations in payback time, ROI, investment and operating costs, and total revenues by varying the prices of PEGMA purchased. 

As can be seen in Figure 9, when the PEGMA price doubled from the base case value of $3,000/t to $6000/t, the payback time increased from 2.37 to 3.45 years (increase of 46%), the ROI reduced by 31% to 28.99%, and the operating cost increased by 34% to $15.94 million/y. When the PEGMA price was increased by 250%, to $10,500/t, the payback time increased exponentially, reaching 10 years. ROI linearly decreased with the increased material cost and it reached 10% at PEGMA price of $10,500/t. The operating cost of the plant increased with the increase of PEGMA cost, and it almost doubled at $22.01 million/y. However, both total capital investment and revenues were insignificantly affected.

##### Purchase Price of WPC

**Figure 10:**
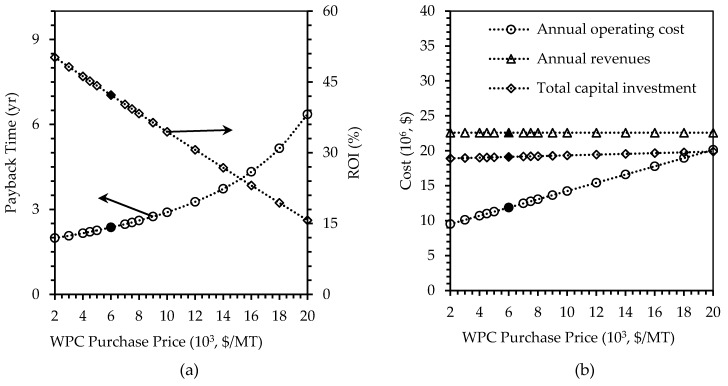
Effects of changing WPC purchase price, on (**a**) payback time in years and ROI in percent, and (**b**) annual operating cost, annual revenues, and total capital investment in U.S. dollars. Filled markers represent results of the base case scenario.

The tornado chart (Figure 3) showed that WPC purchase prices was sensitive to the overall profitability of the project determined by its payback time. This is because large amount of WPC was used, and 45% of the material costs was contributed by WPC, as indicated in Figure 2. The baseline price of WPC ($6000/t) was based off the bulk WPC price taken from USDA Agricultural Marketing Service [16]. By doubling this price to $12,000/t, the payback time and operating cost of the plant increased by 37% and 29.7%, respectively, reaching 3.27 years, and $15.43 million/y. The ROI decreased by 38% to 30.63%. However, changing WPC purchase price did not affect the revenues and capital investment. Given these results, the WPC purchase price can influence the profitability of the plant by almost 35% in all parameters considered (Figure 10). 

Sensitivity analyses thus showed that the profitability of the plant could be affected by changing certain variables associated with the plant’s internal and external factors. 

Future studies may include techno-economic assessment of production of whey-based plastics via other polymerization methods and analyzing different parameters for sensitivity analyses. Moreover, the environmental assessment of such bio-based plastics, including variation in the amount of copolymer and its effects on biodegradability, may also be studied.

## 4. Conclusions

In this study, the techno-economic assessment was conducted for plastic production from whey protein. The feasibility of the production was demonstrated using the SuperPro Designer software for two different scenarios, namely feedstocks of waste whey from the dairy industry (Scenario 1) and WPC purchased from the market (Scenario 2), both through copolymerization reaction with PEGMA. Both scenarios showed profitable outcomes, with payback times of less than five years. Using WPC is more profitable, especially if the plants are employed in non-major dairy-producing countries. A sensitivity analysis was also carried out to account for uncertainties and to determine the fluctuations in the results with changes in different parameters. This study shows that it is economically feasible to produce plastics from natural and biodegradable sources like whey proteins, thereby reducing the wastes associated with dairy products, in addition to utilizing alternatives to fossil-based plastics. 

## Figures and Tables

**Table 1:** Information provided on the equipment necessary for processing of whey and production of plastic in the models analyzed.

Steps	Equipment	Unit Operation	Characteristics
A. Pretreatment of Whey	P-1/PZ-101	Pasteurization	Inlet of 100 t of whey Maximum throughput of 100,000 L/h Pasteurize (61 °C for 15 secs, outlet temp. 30 °C) Heating agent: steam Cooling agent: chilled water
	P-2/DC-101	Centrifugation	Equipment design based on Solids Removal (Carbs 70%, Fats 60%, Minerals 70%, Proteins 100%) Centrifuge for 30 min to remove solids Sedimentation efficiency: 30%
B. Production of WPC	P-3/UF-101 (6 units)	Ultrafiltration	Rejection coefficients: Carbohydrates 0.01 Fats 0.01 Minerals 0.01 Proteins 0.95 Concentration factor (Feed/Retentate): 20 Max. solids conc. in retentate: 600 g/L Solids conc. in retentate: 491 g/L Max. membrane area: 80 m^2^ Membrane area: 76 m^2^ Dft membrane
	P-4/DF-101	Diafiltration	Rejection coefficient: Proteins 0.95 Filtrate flux: 20 L/m^2^hr Max. solids conc. in retentate: 600 g/L Solids conc. in retentate: 340.23 g/L Membrane area: 30.1 m^2^
	P-5/SDR-101	Spray Drying	Volatile component evaporation rate: water 93% Outlet gas and product temp: 70 °C Max. diameter: 10 m Height/Diameter ratio: 3
	P-6/SL-101	Solids Storage	Volume < 1500 L Store WPC powder to partially sell and partially use for following plastic production process
C. Chemical Processing	P-7/R-101	Protein Dissolution	Stirred reactor Max. volume 40,000 L Transfer in 1 t of WPC Charge 10,000 L of water Agitation Time: 2 h (dissolve protein) Charge 100 L of NaOH Agitation Time: 20 min (raise pH to 10~11) Final temp: 26 °C
	P-8/R-102	Methacrylation	Stirred reactor Charge methacrylic anhydride 50 L Agitation Time: 7 h Charge NaOH: 125 L (to raise pH to about 9 so proteins don’t precipitate due to presence of methacrylic acid formed from reaction) Temp: 25 °C
	P-9/R-103	Polymerization	Stirred reactor Max. volume 40,000 L Charge PEGMA 2300 L Charge APS 92 L Charge TEMED 4.6 L Reaction Time: 1 h Temp: 25 °C
D. Production of plastic	P-10/WSH-101 (2 units)	Washing	Wash out excess chemicals Process time: 30 min
	P-11/DC-102	Centrifugation	Equipment design based on Solids Removal (Polymerized content 90%) Centrifugation time: 4 h Sedimentation efficiency: 30% Limiting solid particle minimum diameter: 1 micron
	P-12/DDR-101	Drum Drying	Drum area: 12 m^2^ Evaporate 85% of water Heating agent: steam Drying rate: 40 kg/m^2^hr Drying time: 360 min Final solids temperature: 72 °C

**Table 2:** Results of economic analysis.

Parameter	Scenario 1	Scenario 2
Annual Operating Time (h)	7909.8	7918.1
Unit Production Ref. Rate (t MP^1^/y)	3216.3	3227.2
Batch Size (kg MP)	5469.9	5469.9
Recipe Batch Time (h)	52.4	34.4
No. of Batches (/y)	588	590
Total Capital Investment ($)	33,563,000	19,132,000
Operating Cost ($/y)	12,375,000	11,889,000
Total Revenues ($/y), from:	22,538,000	22,591,000
Plastic stream (MP)	22,514,000	
WPC Powder	23,520	
Cost Basis Annual Rate (t MP/y)	3216.3	3227.3
Unit Production Cost ($/t MP)	3850	3680
Unit Production Revenue ($/t MP)	7010	7000
Gross Margin (%)	45.1	47.4
Net Profit ($/y)	9,087,000	8,082,000
Return On Investment (ROI) (%)	27.1	42.2
Payback Time (y)	3.69	2.37

^1^MP is the main product, i.e., total flow of plastic stream.

**Table 3:** Economic effects of different protein/poly(ethylene glycol) methyl ether methacrylate (PEGMA) proportions (copolymerization effect), at 30:70 vs. 20:80 ratios, for both Scenario 1 and Scenario 2.

Scenario	Protein:PEGMA	Payback Time (yrs)	ROI (%)	Total Capital Investment ($)	Operating Cost ($/y)	Revenues ($/y)
1	30:70	3.69	27.1	33,563,000	12,375,000	22,538,000
	20:80	5.56	17.99	34,254,000	17,285,000	22,538,000
2	30:70	2.37	42.24	19,132,000	11,889,000	22,591,000
	20:80	3.85	25.95	19,789,000	16,787,000	22,545,000

## Supplementary Materials

The following are available online at https://www.mdpi.com/2073-4360/12/4/847/s1, Table S1: Calculation of the minimum selling price (MSP) of the plastics, Table S2: Annual Operating Cost (2019 prices)-Process Summary, Table S3: Profitability Analysis (2019 prices).
