# Supplementary material for: Techno-Economic Assessment of Whey Protein-Based Plastic Production from a Co-Polymerization Process
Source: Polymers (Basel). 2020 Apr 7;12(4):847. doi: 10.3390/polym12040847 (PMC7240497; doi:10.3390/polym12040847)
Supplement: Supplementary file 1 [file polymers-12-00847-s001.pdf]

Supplementary Material

Minimum selling price (MSP):

This is based on breakeven concept in business and economics and the MSP can be calculated as per Eq. S1:

$$\text{Minimum Selling Price} = \frac{\text{Total fixed costs}}{\text{Volume of production}} + \text{Variable cost per unit} \quad \text{(S1)}$$

Payback time:

The payback time is a measure of the time needed for the total capital investment to be exactly balanced by the cumulative net profits. It is calculated by dividing the total capital investment charged to this project by the annual net profit, as per Eq. S2:

$$\text{Payback time (years)} = \frac{\text{Total capital investment}}{\text{Annual net profit}} \quad \text{(S2)}$$

Return on investment (ROI):

The ROI is another profitability measure used to evaluate the viability of an investment or to compare the profitability of a number of different investments. It is calculated by dividing the annual net profit by the total capital investment charged to this project, as shown in Eq. S3:

$$\text{Return on investment, ROI (\%)} = \frac{\text{Annual net profit}}{\text{Total capital investment}} \times 100 \quad \text{(S3)}$$

Results

Table S1. Calculation of the minimum selling price (MSP) of the plastics

	**Scenario 1**	**Scenario 2**
A. Total investment (total fixed capital) ($)	33,563,000	19,132,000
B. Volume of Production per year (t)	3,216.32	3,227.26
C. Unit Production Cost ($/t)	3,850	3,680
Minimum selling price ($/t) = (A/B)+C	14,285.21	9,608.24

Table S2. Annual Operating Cost (2019 prices) - Process Summary

	Scenario 1		Scenario 2	
Cost Item	$	%	$	%
Raw Materials	4,324,000	34.94	7,876,000	66.25
Labor-Dependent	1,034,000	8.36	340,000	2.86
Facility-Dependent	5,923,000	47.86	3,326,000	27.98
Laboratory/QC/QA	155,000	1.25	51,000	0.43
Consumables	565,000	4.57	0	0
Waste Treatment/Disposal	263,000	2.13	264,000	2.22
Utilities	110,000	0.89	33,000	0.28
Transportation	0	0	0	0
Miscellaneous	0	0	0	0
Advertising/Selling	0	0	0	0
Running Royalties	0	0	0	0
Failed Product Disposal	0	0	0	0
TOTAL	12,375,000	100.00	11,889,000	100.00

Table S3. Profitability Analysis (2019 prices)

	Scenario 1	Scenario 2
A. Direct Fixed Capital ($)	31,468,000	17,484,000
B. Working Capital ($)	522,000	774,000

C. Startup Cost ($)	1,573,000	874,000
D. Up-Front R&D ($)	0	0
E. Up-Front Royalties ($)	0	0
F. Total Investment (A+B+C+D+E) ($)	33,563,000	19,132,000
G. Investment Charged to This Project ($)	33,563,000	19,132,000
H. Revenue/Savings Rates		
Plastic (Main Revenue) (kg/y)	3,216,323	3,227,263
WPC powder (Revenue) (kg/y)	23,520	N/A
I. Revenue/Savings Price		
Plastic (Main Revenue) ($/kg)	7	7
WPC powder (Revenue) ($/kg)	1	N/A
J. Revenue/Savings Price		
Plastic (Main Revenue) ($/y)	22,514,263	22,590,843
WPC powder (Revenue) ($/y)	23,520	N/A
1 Total Revenues ($/y)	22,537,783	22,590,843
2 Total Savings ($/y)	0	0
K. Annual Operating Cost (AOC)		
1 Actual AOC ($/y)	12,375,000	11,889,000
2 Net AOC (K1-J2) ($/y)	12,375,000	11,889,000
L. Unit Production Cost /Revenue		
Unit Production Cost ($/kg MP)	3.85	3.68
Net Unit Production Cost ($/kg MP)	3.85	3.68
Unit Production Revenue ($/kg MP)	7.01	7
M. Gross Profit (J-K) ($/y)	10,163,000	10,702,000
N. Taxes (40%) ($/y)	4,065,000	4,281,000
O. Net Profit (M-N + Depreciation) ($/y)	9,087,000	8,082,000
Gross Margin (M/J1 x100) (%)	45.09	47.37
Return On Investment (O/F×100) (%)	27.08	42.24
Payback Time (F/O) (y)	3.69	2.37
